# Supplementary material for: Altered microRNA Transcriptome in Cultured Human Liver Cells upon Infection with Ebola Virus
Source: Int J Mol Sci. 2021 Apr 6;22(7):3792. doi: 10.3390/ijms22073792 (PMC8038836; doi:10.3390/ijms22073792)
Supplement: Supplementary file 1 [file ijms-22-03792-s001.zip › Supplementary_File/D_ KEGG_Analysis_Results/16-30nt_pathway_Makona-96h-Huh7_vs_Control-96h-Huh7_up.mature_mirna_targets/hsa_EnrichmentScoreDotPlot.pdf]

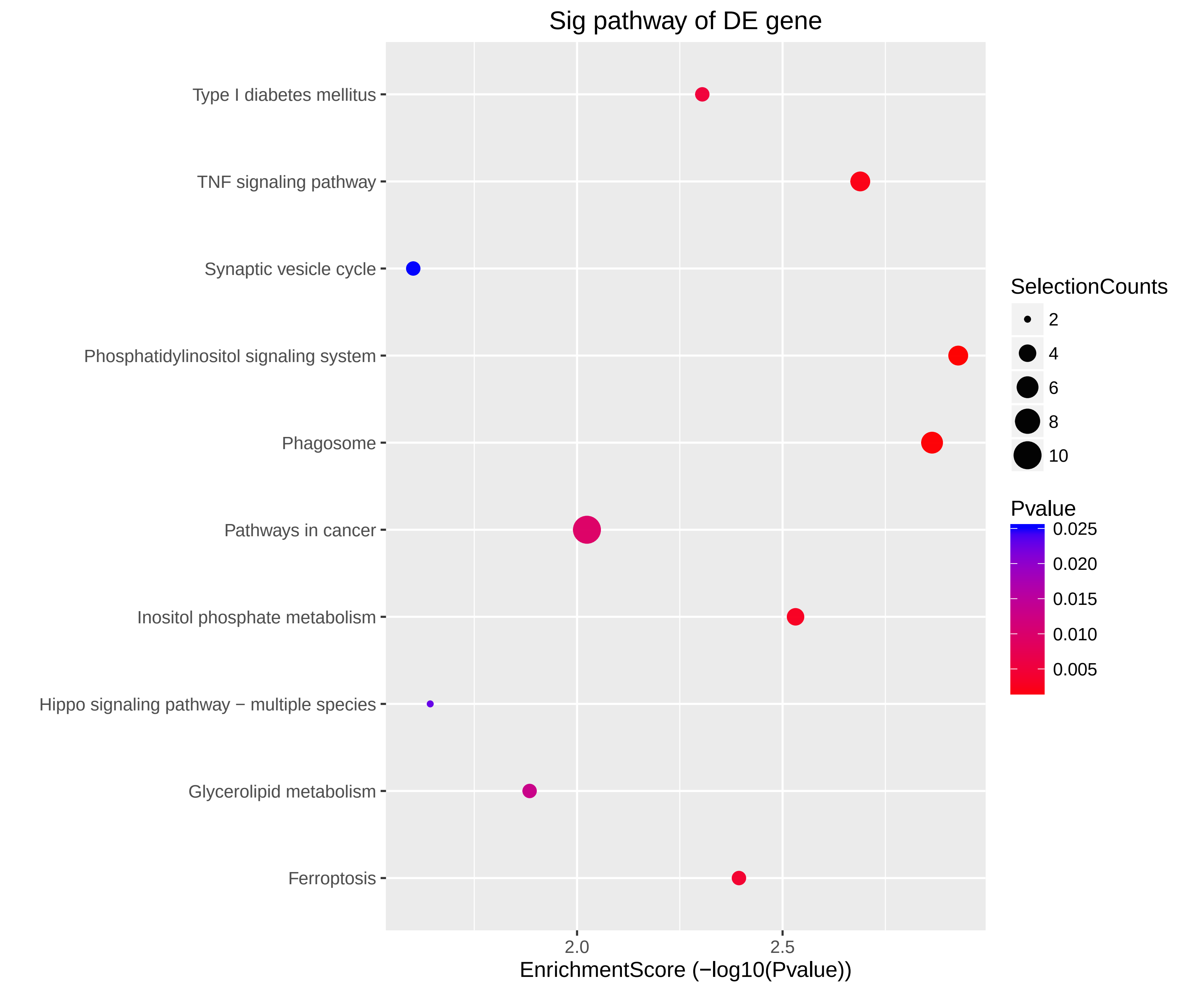

Sig pathway of DE gene
Type I diabetes mellitus
TNF signaling pathway
Synaptic vesicle cycle
Phosphatidylinositol signaling system
Phagosome
Pathways in cancer
Inositol phosphate metabolism
Hippo signaling pathway − multiple species
Glycerolipid metabolism
Ferroptosis
2.0
2.5
EnrichmentScore (−log10(Pvalue))
SelectionCounts
2
4
6
8
10
Pvalue
0.025
0.020
0.015
0.010
0.005
